# Feature selection in wind speed forecasting systems based on meta-heuristic optimization

**DOI:** 10.1371/journal.pone.0278491

**Published:** 2023-02-07

**Authors:** El-Sayed M. El-kenawy, Seyedali Mirjalili, Nima Khodadadi, Abdelaziz A. Abdelhamid, Marwa M. Eid, M. El-Said, Abdelhameed Ibrahim

**Affiliations:** 1 Department of Communications and Electronics, Delta Higher Institute of Engineering and Technology, Mansoura, Egypt; 2 Centre for Artificial Intelligence Research and Optimization, Torrens University Australia, Fortitude Valley, QLD, Australia; 3 Yonsei Frontier Lab, Yonsei University, Seoul, South Korea; 4 Department of Civil & Environmental Engineering, Florida International University, Miami, FL, United States of America; 5 Department of Computer Science, Faculty of Computer and Information Sciences, Ain Shams University, Cairo, Egypt; 6 Department of Computer Science, College of Computing and Information Technology, Shaqra University, Shaqra, Saudi Arabia; 7 Faculty of Artificial Intelligence, Delta University for Science and Technology, Mansoura, Egypt; 8 Electrical Engineering Department, Faculty of Engineering, Mansoura University, Mansoura, Egypt; 9 Delta Higher Institute of Engineering and Technology (DHIET), Mansoura, Egypt; 10 Computer Engineering and Control Systems Department, Faculty of Engineering, Mansoura University, Mansoura, Egypt; Karlstad University: Karlstads Universitet, SWEDEN

## Abstract

Technology for anticipating wind speed can improve the safety and stability of power networks with heavy wind penetration. Due to the unpredictability and instability of the wind, it is challenging to accurately forecast wind power and speed. Several approaches have been developed to improve this accuracy based on processing time series data. This work proposes a method for predicting wind speed with high accuracy based on a novel weighted ensemble model. The weight values in the proposed model are optimized using an adaptive dynamic grey wolf-dipper throated optimization (ADGWDTO) algorithm. The original GWO algorithm is redesigned to emulate the dynamic group-based cooperative to address the difficulty of establishing the balance between exploration and exploitation. Quick bowing movements and a white breast, which distinguish the dipper throated birds hunting method, are employed to improve the proposed algorithm exploration capability. The proposed ADGWDTO algorithm optimizes the hyperparameters of the multi-layer perceptron (MLP), K-nearest regressor (KNR), and Long Short-Term Memory (LSTM) regression models. A dataset from Kaggle entitled Global Energy Forecasting Competition 2012 is employed to assess the proposed algorithm. The findings confirm that the proposed ADGWDTO algorithm outperforms the literature’s state-of-the-art wind speed forecasting algorithms. The proposed binary ADGWDTO algorithm achieved average fitness of 0.9209 with a standard deviation fitness of 0.7432 for feature selection, and the proposed weighted optimized ensemble model (Ensemble using ADGWDTO) achieved a root mean square error of 0.0035 compared to state-of-the-art algorithms. The proposed algorithm’s stability and robustness are confirmed by statistical analysis of several tests, such as one-way analysis of variance (ANOVA) and Wilcoxon’s rank-sum.

## 1 Introduction

A long-term energy supply can be delivered using wind energy and thus plays a crucial role in micro-grid intelligent grid architecture as an essential low-carbon energy source. The increased utilization of wind power in power grids might substantially influence system reliability and quality, given that the generated amount of wind energy is directly proportional to the wind speed. A precise wind speed forecasting technology can improve the safety and stability of power systems [1].

Wind power generation, on the other hand, is inherently unpredictable and intermittent, providing several challenges to broader adoption. With the aid of wind speed and power generation estimations, it is possible to reduce energy balance and make production scheduling and dispatching decisions. Furthermore, projections can lower costs by anticipating demand for wind curtailments and increasing profits in power market operations. However, reliable forecasting of wind speed and power is exceedingly difficult due to the wind’s unstable and unpredictable nature. A wind power prediction estimates the production of one or more wind turbines, referred to as wind farms. Forecasts may also be expressed in energy by combining power production across each period [2].

The fundamental objective of wind speed and power forecasting is to provide vital information regarding the expected wind power and speed for the following days, hours, or minutes. The four-time frames that can be classified according to power system operation needs are long-term (from seven days down to one day), medium-term (from twenty-four hours down to six hours), short-term (from six hours down to thirty minutes), and highly short-term (from thirty minutes down to a few seconds). Control of turbines and load tracking are reliant on extremely short-term forecasts. It is possible to distribute preloads using short-term forecasting. Medium-term projections are utilized for both power system management and energy trading. Using long-term estimates, maintenance strategies for wind turbines are developed [3].

The forecasting of wind speed is a time-sensitive and non-linear challenge, which motivates researchers to utilize the information contained in previous wind data. Long short-term memory (LSTM) networks, which are based on time-series data, are one of the most popular methods for making predictions [4]. Utilizing statistical and numerical weather prediction models, the topic of wind power forecasting was discussed. Two locations in Brazil leverage Brazilian advances in the regional atmospheric modeling system to simulate wind speed estimates 72 hours in advance, every ten minutes [5].

Based on a dataset of two months of recordings and fifteen minutes of sampling, authors in [6] forecast the wind power of sixteen wind farms in China based on a back-propagation neural network (BPNN), least squares support vector machine, and radial basis function NN. Authors in [3] applied deep learning NN and isolation forest (IF) to predict the wind power using SCADA data from a wind turbine located in Scotland, with a one-second sampling rate of a 12-month dataset. In Scotland, a seven-megawatt wind turbine is monitored by utilizing a twelve-month dataset with a one-second sample rate and an IF and feed-forward NN.

The authors of [7] used restricted Boltzmann machines and rough set theory to create an interval probability distribution learning (IPDL) model for capturing the unsupervised temporal characteristics of wind speed data. The IPDL model collects interval-adjustable latent variables in order to capture the probability distribution of wind speed time-series data. A real-valued interval deep belief network (IDBN) for supervised regression of future wind speed data was developed using the IPDL model and fuzzy type II inference system. Deep neural network (DNN) architecture with stacked denoising auto-encoder (SDAE) and stacked auto-encoder was created for wind speed forecasting by the creators of [8].

Wind speed time series forecasts were generated using the temporal features retrieved from the network nodes. The authors presented deep convolutional learning (GCDL) in [9] as a scalable framework for learning strong Spatio-temporal characteristics from nearby wind farms using wind direction and speed data. Their model included the rough set theory and the GCDL architecture. Authors in [10] provided a framework for improving the architecture and hyperparameters of the LSTM deep learning model for predicting wind speed based on an upgraded grasshopper optimization method.

To predict short-term wind speed, researchers in [11] used wavelet transform variants and a variety of support vector regression (SVR). To find the optimal regressor for wind forecasting applications, they tested their suggested methodologies using a variety of performance metrics. Random forests, convolutional neural networks (CNN), discrete wavelet transform (DWT), and Twin SVR were utilized by authors in [12] for wind forecasting. The wavelet transform was used to improve the information retrieved from wind speed. In addition, authors in [13] developed an adaptive threshold and twin SVM (TWSVM) approach for detecting the anomaly problem in wind turbine gearboxes. Among the most modern methods for predicting wind power are shown in Table 1.

**Table 1:** Recent research approaches for wind power forecasting, m and s indicate minutes and seconds, respectively.

Reference	Methodology	Dataset	Dataset size	Sampling rate
[5]	Statistical regression, calman filter	Two wind farms in Brazil	7 & 12 months	10 m
[6]	LLSSVM, RBFNN, and BRPNN	16 MW wind farm in China	2 months	15 m
[14]	MODA optimzed ELM	Two wind farms in China	37 days	10 m
[15]	MLP	Several farms in Senegal	6 to 9 months	1 & 10 m
[9]	RS, LSTM, GCDLA	145 wind farms in US	6 years	5 m
[7]	Rough set, Boltzman Machines, IDBN, IPDL	A farm in US	3 years	10 m
[16]	GMM, LSTM	123 wind farms in China	3 months	15 m
[17]	DGF, RBFNN, CNN	A wind farm in Taiwan	12 months	60 m
[3]	Feed forward NN, Isolation forest (IF)	Seven MW wind farms in Scotland	12 months	1 s
[10]	Advanced grasshopper optimization, LSTM	Two farms in US	12 months	30 m
[18]	AD-PSO-Guided WOA, LSTM	Kaggle Global Energy Forecasting	18 months	60 m

The authors of [14] proposed a novel framework for electrical power system forecasting based on MODA (multi-objective dragonfly algorithm). In this study, the MODA method was used to optimize a modified Elman neural network (ENN) model. The tested dataset was collected at two observation locations in Penglai, China, over the course of 37 days, at a sampling rate of 10 minutes per site. In [15], the authors proposed an Artificial Neural Network-based wind turbine power output forecast model (ANN). Their data was collected at three additional sites along the northwest coast of Senegal between 6 and 9 months, with a sampling rate between 1 and 10 minutes for each sample. Inspired by the localized first-order approximation of spectral graph convolutions, a scalable graph convolutional deep learning architecture (GCDLA) employs extracted temporal features to predict the wind-speed time series of the complete network nodes [9]. The simulation findings based on 145 wind stations in the Northern States of the United States for six years with a sampling rate of five minutes demonstrate the benefits of capturing spatial and temporal interval information at a deep level.

On the basis of LSTM and the Gaussian mixture model (GMM), short-term forecasting and uncertainty analysis of wind turbine output were provided using a dataset of 123 wind farm units in north China for three months with a 15-minute sample rate [16]. In [17], on the basis of a Taiwanese wind farm, a hybrid deep learning-based neural network for 24-h wind power forecasting with a 60-minute sample rate is presented. The authors of [18] proposed ensemble wind speed forecasting utilizing deep learning and an adaptive dynamic optimization algorithm with a sample rate of 60 minutes for 18 months.

In order to improve wind speed forecasts, this research makes use of a novel optimization algorithm that is referred to as ADGWDTO. This algorithm is built on the grey wolf and dipper throated optimization techniques. Even though it is easy to use and strikes an excellent balance between exploration and exploitation, Grey Wolf Optimization (GWO) [19] has a few drawbacks, including a low exploration rate and a performance drop when there are a lot of different local optimum solutions. These issues arise when there are a lot of different possible solutions. The performance of the Dipper Throated Optimization (DTO) [20] method deteriorates due to the fact that it is dependent on a large number of variables during the optimization process. In addition to this, the algorithm’s convergence has been achieved too soon. However, a significant advantage is presented by the fact that there is a healthy equilibrium between exploration and utilization. The DTO algorithm is used in the suggested strategy so that users can make use of the benefit that is offered. This research makes use of the dipper throated optimizer, which is an algorithm, to make the most of the benefits offered by this method while also accounting for its limits.

Therefore, the purpose of this research is to provide a brand new ensemble model that utilizes an innovative meta-heuristic optimization approach in order to make forecasts regarding the speed of the wind. The suggested ensemble model is made up of three different machine learning regression models. These models are the multi-layer perceptron (MLP), the k-nearest regressor (KNR), and the long short-term memory (LSTM). Utilizing the proposed novel optimization method that is referred to as ADGWDTOO and is based on the grey wolf and dipper throated optimization algorithms results in an improvement in the performance of the proposed ensemble model. In order to make accurate forecasts of wind speed, the ADGWDTO method that was recently developed is used to optimize the hyper-parameters of the regression models as well as the weighted ensemble model. A dataset taken from the Kaggle global energy forecasting competition [21] is used to predict the hourly power output up to 48 hours in advance. This is done so that the effectiveness of the methodology that has been proposed may be evaluated.

The feature selection process of the wind power forecasting dataset is solved using a new binary-based ADGWDTO algorithm. Compared to algorithms, such as Genetic Algorithm (GA) [22], Firefly Algorithm (FA) [23], Particle Swarm Optimization (PSO) [24], Whale Optimization Algorithm (WOA) [25–27], Grey Wolf Optimizer (GWO) [19], Dipper Throated Optimization (DTO) [20], the proposed algorithm is confirmed to achieve the best performance. In addition, comparisons are made between the proposed ensemble model and three other ensemble models to demonstrate its superiority and efficacy. ANOVA and Wilcoxon’s rank-sum tests are conducted to validate the accuracy of the proposed methods.

The following is an explanation of the primary contributions made by this work:

- In this paper, we propose a brand new adaptive dynamic grey wolf-dipper throated optimization (ADGWDTO) technique.
- In order to choose features from the dataset that was put through testing, a binary ADGWDTO method, which is a binary variant of the suggested technique, is used.
- AIn order to enhance the accuracy of the tested dataset’s classification, a weighted optimal ensemble model has been built. This model is based on the ADGWDTO technique that was proposed.
- The Wilcoxon rank-sum test and the ANOVA test are used to evaluate the statistical significance of the ADGWDTO algorithm.
- The ADGWDTO algorithm is used to improve the performance of classification methods for the goals of classifying data so that it can be used in new applications.
- Both the binary ADGWDTO technique and the classification algorithm that is based on regression models can be generalized and evaluated for a wide variety of datasets.

The paper structure for the subsequent sections is as follows: The literature review for the procedures and materials is presented in Section 2. The methods proposed are then detailed in section 3. Section 4 presents and analyzes the experimental outcomes. In Section 5, the conclusions and future directions are presented.

## 2 Methods and materials

This section will examine MLP, KNR, and LSTM fundamental models. The ensemble model technique will also be introduced to illustrate how it works with fundamental models. The methods for adaptive grey wolf optimization and dipper throated optimization will also be described.

### 2.1 Multi-layer perceptron (MLP)

Several artificial neural networks (ANNs) can be utilized for classification and prediction. ANNs can simulate the discovery of data patterns or sets of cause-and-effect variables by employing transient detection, approximation, time-series forecasting, and pattern recognition approaches. In ANNs, a group of neurons are densely connected and operate together to solve regression and classification problems in various fields [28]. MLP is a type of ANNs in which neurons are organized in the form of layers referred to as input, hidden, and output layers. The weighted sum of a neuron’s output value is computed as follows [29]:

$$\begin{array}{c} S_{j}=\sum_{i=1}^{n}w_{ij}I_{i}+\beta_{j} \end{array}$$ (1)

where *I*_*i*_ is an input, *w*_*ij*_ is neuron *j* and input *I*_*i*_ connection weight. *β*_*j*_ is the bias value. The output of a neuron *j* can be calculated as follows:

$$\begin{array}{c} f_{j}\left(S_{j}\right)=\frac{1}{1+e^{-S_{j}}} \end{array}$$ (2)

where a sigmoid function is used, and the *f*_*j*_(*S*_*j*_) value can be used to get the output of the network as

$$\begin{array}{c} y_{k}=\sum_{j=1}^{m}w_{jk}f_{j}\left(S_{j}\right)+\beta_{k} \end{array}$$ (3)

where *w*_*jk*_ represents the output node *k* and hidden layer neuron *j* weight. *β*_*k*_ refers to the output layer bias value.

### 2.2 K-Nearest Neighbor Regressor (KNR)

Using the utilized distance measures, KNR depends on historical occurrences that are most comparable to the current state in order to make predictions. Predictions are generated using a weighted average based on the *K* nearest neighbors. KNR uses Euclidean distance as a metric to measure a distance between *X*_*train*_ and *X*_*t*_*est* sets as follows.

$$\begin{array}{c} D\left(x_{train,i},x_{test,i}\right)=\sqrt{\sum_{i=1}^{k}{\left(x_{train,i},x_{test,i}\right)}^{2}} \end{array}$$ (4)

The prediction results of the test data are generated using the following equation:

$$\begin{array}{c} \hat{y}=\sum_{j=1}^{k}\left(w_{j}y_{j}\right) \end{array}$$ (5)

where *w*_*j*_ refers to the weight of the *j*th neighbor. The value of this weight is adjusted using the observed data. For the number of training data denoted by *n*, the value of *w*_*j*_ is measured as *w*_*j*_ = *j*/*n*.

### 2.3 Long short term memory (LSTM)

**Fig 1:**
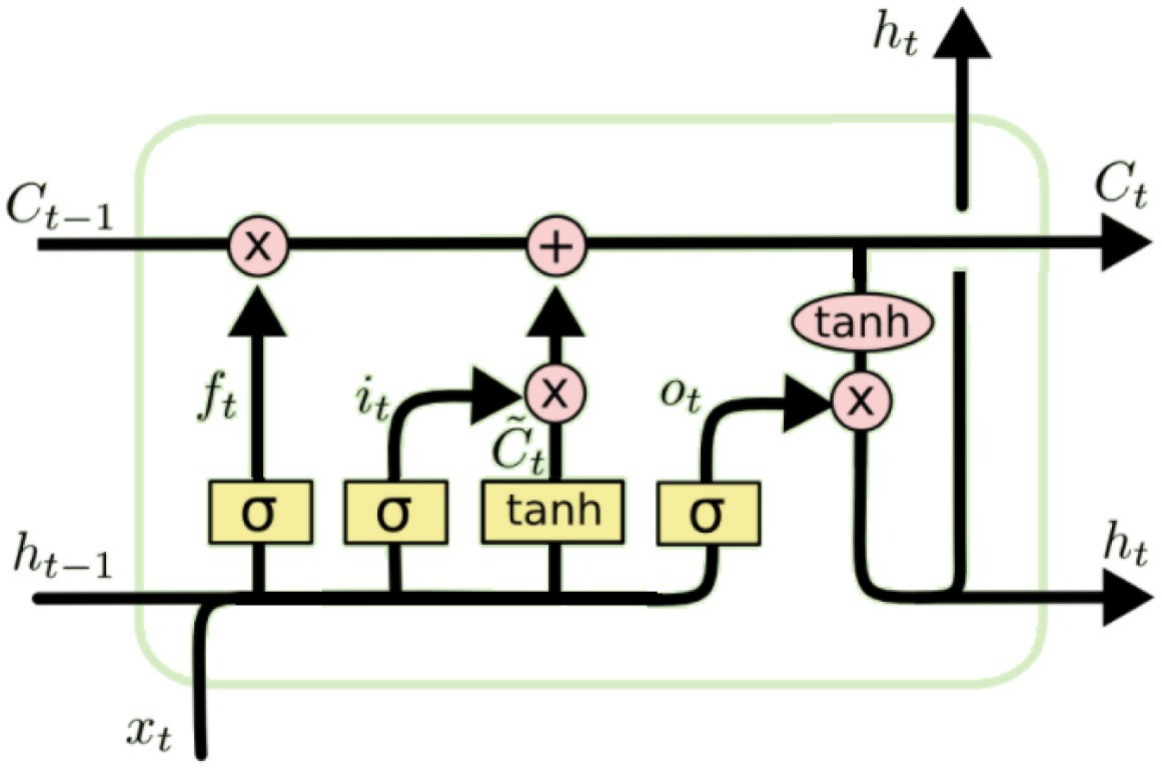
The LSTM model structure.

According to [30], LSTM is an improved ANN model that may be used to solve various issues. The key benefit of the LSTM is its ability to retain information over an extended period. Fig 1 depicts the LSTM design in all its nifty glory. Decisions on which cell state data to reject are made in the LSTM model’s initial phase. Eq 6 describes the usage of a sigmoid layer for this purpose.

$$\begin{array}{c} f_{t}=\sigma \left(b_{f}+W_{f}\left[h_{t-1},x_{t}\right]\right) \end{array}$$ (6)

In the next stage, the cell state will be updated with new input data. New candidates are selected by the sigmoid layer and added to the produced state in Eqs 7 and 8 as shown in this section.

$$\begin{array}{c} i_{t}=\sigma \left(b_{i}+W_{i}\left[h_{t-1},x_{t}\right]\right) \end{array}$$ (7)

$$\begin{array}{c} {\bar{C}}_{t}=tanh\left(b_{i}+W_{i}\left[h_{t-1},x_{t}\right]\right) \end{array}$$ (8)

The cell previous state denoted by *C*_*t*−1_ parameter is then updated to a new state referred to as *C*_*t*_ parameter in Eq 9 based on Eqs 6–8.

$$\begin{array}{c} C_{t}={\bar{C}}_{t}\times i_{t}+C_{t-1}\times f_{t} \end{array}$$ (9)

The final stage is to make a choice regarding the final product. It is the sigmoid layer’s job to determine which cell state portions should be outputted. The sigmoid gate output is then multiplied by tanh and force values between [−1, 1] in the cell state.

$$\begin{array}{c} h_{t}=o_{t}\times tanh\left(C_{t}\right),o_{t}=\sigma \left(b_{o}+W_{o}\left[h_{t-1},x_{t}\right]\right) \end{array}$$ (10)

### 2.4 Ensemble models

The basic objective of ensemble models is to combine the capabilities of multiple individual base models into a unified model with enhanced performance. Several methods can be followed to realize this approach of ensemble models. Resampling the training set, for example, serves as an effective strategy, while other techniques use different prediction methods or modify specific parameters of a predictive model [31]. This article proposes a weighted ensemble model composed of three machine learning models, MLP, KNR, and LSTM. The weights of the ensemble model are optimized using a new optimization approach discussed in the following sections. On the other hand, other ensemble models, such as average and SVR ensemble, are used in the experiments conducted to show the proposed ensemble’s effectiveness.

### 2.5 Adaptive grey wolf optimization

Despite its widespread usage in optimization, the original GWO has been shown to have several shortcomings and limitations. These downsides include early convergence, limited precision, and an inability to locate the ideal solution. You can easily get stranded and locked in the local optima, created by wolves’ leader alpha, beta, and gamma, all converging to the same solution. This can be pretty dangerous. As a result, the three leaders constantly change their positions in response to each other. The GWO’s capacity to organize and handle the complicated search space is limited. A further problem with this design is the inability of GWO to properly balance exploratory work with operational work as experimental is carried out first, and functional work is carried out second. To put things into perspective, getting out of the local optima in the final GWO iteration would be a challenge and an impediment. As a result, the search for an optimal answer may become empty. In addition, the GWO algorithm’s performance is strongly impacted by the number of variables, which is attributable to the initial population of a local solution.

The original GWO algorithm is redesigned in this work to emulate the dynamic group-based cooperative to address the difficulty of establishing the balance between exploration and exploitation. There are three solutions in the grey wolf optimization: alpha (**S**_*α*_) which is the best solution, followed by beta (**S**_*β*_), delta (**S**_*δ*_). The other solutions retrieved by the algorithm are denoted by (**S**_*γ*_). The following is the formulation of the grey wolf optimization.

$$\begin{array}{c} S\left(t+1\right)=S_{p}\left(t\right)-A.\left|C.S_{p}\left(t\right)-S\left(t\right)\right| \end{array}$$ (11)

where **S** represents the agent position, and *t* is the current iteration. **S**_*p*_(*t*) is the best agent (prey) position and **A** and **C** are defined as follow.

$$\begin{array}{c} A=2a.r_{1}-a \end{array}$$ (12)

$$\begin{array}{c} C=2r_{2} \end{array}$$ (13)

where **r**_1_ and **r**_2_ are randomly selected values in [0, 1], and **a** is selected in [0, 2] with a linearly decreasing. To control the balance between the exploitation and exploration, the value of **a** is updated as follows based on the available iterations *M*_*t*_.

$$\begin{array}{c} a=2-t.\frac{2}{M_{t}} \end{array}$$ (14)

The process of agent position updating is described using the following equations based on the three fittest solutions, **S**_*α*_, **S**_*β*_, and **S**_*δ*_.

$$\begin{array}{c} S_{1}=S_{\alpha }-A_{1}.D_{\alpha },\;D_{\alpha }=\left|C_{1}.S_{\alpha }-S\right|\\ S_{2}=S_{\beta }-A_{2}.D_{\beta },\;D_{\beta }=\left|C_{2}.S_{\beta }-S\right|\\ S_{3}=S_{\delta }-A_{3}.D_{\delta },\;D_{\delta }=\left|C_{3}.S_{\delta }-S\right| \end{array}$$ (15)

where **A**_1_, **A**_2_, and **A**_3_ are calculated by Eq (12). **C**_1_, **C**_2_, and **C**_3_ are calculated by Eq (13). The new position of population agents is determined by the following equation.

$$\begin{array}{c} S\left(t+1\right)=\frac{S_{1}+S_{2}+S_{3}}{3} \end{array}$$ (16)

The global minimum finding is a complex undertaking. Two methods to accomplish its task by the GWO: exploration and exploitation. Discovering exciting places in the search space is the process of exploration; on the other hand, finding better spots close to previously successful solutions is the exploitation optimization algorithms benefit from exploration because it keeps them from being stuck in local optimums. Search space exploration is encouraged in the early stages of an optimization algorithm’s development. Finally, in subsequent rounds, agents utilize the knowledge gained to find the global minimum. There were two groups of agents in the adaptive GWO’s population division: group *n*_1_ and group *n*_2_. The GWO is redesigned to emulate the dynamic group-based cooperative to address the difficulty of establishing the balance between exploitation and exploration. Algorithm 1 presents the adaptive GWO algorithm in detail.

**Algorithm 1**: Adaptive GWO.

1: **Initialize** population **S**_*i*_(*i* = 1, 2, …, *n*) with size *n*, fitness function *F*_*n*_, and iterations *M*_*t*_.

2: **Initialize** parameters **a**, **A**, **C**, and *t* = 1

3: **Calculate** *F*_*n*_ for each agent **S**_*i*_

4: **Get** best, second best and third best agents as **S**_*α*_, **S**_*β*_, **S**_*δ*_

5: **while** *t* < *M*_*t*_ **do**

6:  **Update** exploration group (*n*_1_) and exploitation group (*n*_2_) for *n* = *n*_1_ + *n*_2_

7:  **if** (Best *F*_*n*_ is the same for the last three iterations) **then**

8:   **Increase** exploration group agents (*n*_1_)

9:   **Decrease** exploitation group agents (*n*_2_)

10:  **end if**

11:  **for** (*i* = 1 : *i* ≤ *n*_1_) **do**

12:   **Calculate** **S**_1_, **S**_2_, **S**_3_ by Eq 15

13:   **Update** agents’ positions using Eq 16

14:  **end for**

15:  **for** (*i* = 1 : *i* ≤ *n*_2_) **do**

16:   **Calculate** **S**_1_, **S**_2_, **S**_3_ by Eq 15

17:   **Update** agents’ positions using Eq 16

18:  **end for**

19:  **Update** **a** by Eq 14, **A** and **C** by Eqs 12 and 13, *t* = *t* + 1

20:  **Calculate** *F*_*n*_ for each agent **S**_*i*_

21:  **Update** **S**_*α*_, **S**_*β*_, **S**_*δ*_

22: **end while**

23: **Return** **S**_*α*_

### 2.6 Dipper throated optimization

Birds of the Cinclids family are known for their bobbing or dipping motions while perched, such as the Dipper Throated bird. To distinguish a bird from other passerines is to allow it to dive, swim, and hunt below the water’s surface. It charges recklessly into the turbulent or fast-flowing water to catch its prey. Pebbles and stones picked up by the trawler kill little fish and invertebrates that live in the water. The great white shark moves around the ocean floor with the help of its hands. It can dive deep into the water and immerse itself for a long time while utilizing its wings to drive it through the water. In the Dipper-Throated Optimization (DTO) approach, a flock of birds is assumed to swim in search of food [20].

A white breast and quick bowing movements, which distinguish the dipper throated birds hunting method, are employed to improve the proposed algorithm in this work exploration capability. The following matrices represent the locations and velocities of the birds.

$$\begin{array}{c} A=[\begin{array}{ccccc} A_{1,1} & A_{1,2} & A_{1,3} & ... & A_{1,d}\\ A_{2,1} & A_{2,2} & A_{2,3} & ... & A_{2,d}\\ A_{3,1} & A_{3,2} & A_{3,3} & ... & A_{3,d}\\ ... & ... & ... & ... & ...\\ A_{m,1} & A_{m,2} & A_{m,3} & ... & A_{m,d} \end{array}] \end{array}$$ (17)

where *A*_*i*,*j*_ represents the position of *i*^*th*^ bird in the *j*^*th*^ dimension.

$$\begin{array}{c} B=[\begin{array}{ccccc} B_{1,1} & B_{1,2} & B_{1,3} & ... & B_{1,d}\\ B_{2,1} & B_{2,2} & B_{2,3} & ... & B_{2,d}\\ B_{3,1} & B_{3,2} & B_{3,3} & ... & B_{3,d}\\ ... & ... & ... & ... & ...\\ B_{m,1} & B_{m,2} & B_{m,3} & ... & B_{m,d} \end{array}] \end{array}$$ (18)

where *B*_*i*,*j*_ represents the velocity of *i*^*th*^ bird in the *j*^*th*^ dimension. For each agent (bird), the fitness functions values are calculated as follows.

$$\begin{array}{c} h=[\begin{array}{c} h_{1}\left(A_{1,1},A_{1,2},A_{1,3},...,A_{1,d}\right)\\ h_{2}\left(A_{2,1},A_{2,2},A_{2,3},...,A_{2,d}\right)\\ h_{3}\left(A_{3,1},A_{3,2},A_{3,3},...,A_{3,d}\right)\\ ...\\ h_{m}\left(A_{m,1},A_{m,2},A_{m,3},...,A_{m,d}\right) \end{array}] \end{array}$$ (19)

where the fitness score reflects the agent’s quest for food, the superior value indicates the mother bird. In the DTO algorithm, the bird’s position and velocity of the agents are updated as follows for **A**_*best*_ represents the best solution and other birds (follower birds) are indicated as **A**_*nd*_.

$$\begin{array}{c} \begin{array}{c} A\left(t+1\right)=\{\begin{array}{cc} X & \text{if}\;R<0.5\\ Y & otherwise \end{array}, \end{array} \end{array}$$ (20)

where **X** and **Y** are calculated as in the following equations.

$$\begin{array}{c} X=A_{best}\left(t\right)-K_{1}.\left|K_{2}.A_{best}\left(t\right)-A\left(t\right)\right| \end{array}$$ (21)

$$\begin{array}{c} Y=A(t)+B(t+1) \end{array}$$ (22)

where **B**(t + 1) is calculated as

$$\begin{array}{c} \begin{array}{cc} B\left(t+1\right)=K_{3}B\left(t\right) & +K_{4}r_{1}\left(A_{best}\left(t\right)-A\left(t\right)\right)\\ & +K_{5}r_{2}\left(A_{Gbest}-A\left(t\right)\right) \end{array} \end{array}$$ (23)

where **A**_*Gbest*_ indicates the global best solution. *t* is iteration number, and **B**(*t* + 1) represents the agent’s velocity at iteration *i* + 1. **K**_1_, **K**_2_, and **K**_3_ are variable weight values while, **K**_4_, and **K**_5_ are constants. **r**_1_ and **r**_2_ are selected randomly in [0, 1]. The parameters of the classification neural network will be improved using the continuous DTO algorithm, while a binary version of the DTO algorithm is used to select features. The DTO algorithm is explained in Algorithm 2 [20].

**Algorithm 2**: DTO Algorithm.

1: **Initialize** the positions **A**_*i*_(*i* = 1, 2, …, *n*), the velocities **B**_*i*_(*i* = 1, 2, …, *n*), and fitness **h** as in Eqs 17–19.

2: **Initialize** parameters *M*_*t*_, **K**_1_, **K**_2_, **K**_3_, **K**_4_, **K**_5_, **r**_1_, **r**_2_, **R**, *t* = 1

3: **Get** **h** for all agents **A**_*i*_

4: **Find** the best agent **A**_*best*_

5: **while** *t* ≤ *M*_*t*_ **do**

6:  **for** (*i* = 1 : *i* < *n* + 1) **do**

7:   **if** (**R** < 0.5) **then**

8:    **Update** agent position as in Eq 21

9:   **else**

10:    **Update** agent velocity as in Eq 23

11:    **Update** agent position as in Eq 22

12:   **end if**

13:  **end for**

14:  **Get** **h** for all agents **A**_*i*_

15:  **Update** **K**_1_, **K**_2_, **R**, *t* = *t* + 1

16:  **Find** best agent **A**_*best*_

17:  **Set** **A**_*Gbest*_ = **A**_*best*_

18: **end while**

19: **Return** **A**_*Gbest*_

## 3 The proposed methodology

The optimization of the machine learning models and the proposed ensemble models is conducted using the provided and discussed optimization algorithm proposed in this section. The suggested optimization approach is based on the adaptive dynamic grey wolf dipper throated optimization (ADGWDTO) algorithm, which divides the population into two groups, as explained in the following sections. The proposed optimization algorithm’s steps are detailed in Algorithm 3.

### 3.1 Exploration group

This particular group is in charge of the exploration process, which aims to locate potentially fruitful places within the search space. It is also responsible for ensuring that the ADGWDTO does not get stuck in a local optimum and for obtaining the fact that the organization implements two different tactics.

#### 3.1.1 Mutation

It is used to ensure the diversity of the population, which permits the ADGWDTO optimizer to search in various search spaces.

#### 3.1.2 Explore around the solution

The candidate searches in search space around the promising regions surrounding its position in search space by utilizing the following equations to find the optimal fitness.

$$\begin{array}{c} D=r_{1}\left(S\left(t\right)-1\right) \end{array}$$ (24)

$$\begin{array}{c} S\left(t+1\right)=S\left(t\right)+D\left(2r_{2}-1\right) \end{array}$$ (25)

$$\begin{array}{c} \begin{array}{cc} & P\left(t+1\right)=\{\begin{array}{cc} P_{best}\left(i\right)-K_{1}\left|K_{2}P_{best}\left(i\right)-P\left(i\right)\right| & \text{if}\;R<0.5\\ P(i)+V(i+1) & otherwise \end{array} \end{array} \end{array}$$ (26)

**Algorithm 3**: The proposed ADGWDTO algorithm.

1: **Initialize** the population **X**_*i*_(*i* = 1, 2, …, *n*) with size *n*, fitness function **F**_*n*_, and iterations *Max*_*iter*_.

2: **Initialize** parameters **a**, **A**, **C**, *l*, *R*, **r**_1_, **r**_2_, **r**_3_, and *t* = 1

3: **Calculate** fitness function **F**_*n*_ for all agents **G**_*i*_

4: **Find** best solution X*

5: **while** *t* ≤ *Max*_*iter*_ **do**

6:  **D** = **r**_1_(**S**(*t*) − 1)

7:  **S**(*t* + 1) = **S**(*t*) + **D**(2**r**_2_ − 1)

8:  In each group, **Update** the number of solutions

9:  **if** best fitness did not improve fro 3 iterations **then**

10:   **Increase** in the exploration group solutions number

11:   **Mutate** the solution by

$K=1-\frac{2k*Xt^{2}}{Solutions-count^{2}}$

12:   **Update** **r**_1_, **r**_2_, **k**, **z** and **Y**

13:   **Decrease** **k** exponentially from 1 to 0

14:  **end if**

15:  **for** each agent in the exploration group **do**

16:   **Update** **k**_1_, **k**_2_, and **Y**

17:   **if** **Y** < any of the best agents **then**

18:    **Move** towards the best agent by

$P\left(t+1\right)=\{\begin{array}{cc} P_{best}\left(i\right)-K_{1}\left|K_{2}P_{best}\left(i\right)-P\left(i\right)\right| & \text{if}\;R<0.5\\ P(i)+V(i+1) & otherwise \end{array}$

19:   **end if**

20:  **end for**

21:  **for** each agent in the exploitation group **do**

22:   The best solutions were elitism

23:   **Update** **r**_4_, **r**_5_, **k** and **Y**

24:   **D** = **L**(*t*) * (**K** − **r**_4_)

25:   **S**(*t* + 1) = **S**(*t*) + **D**.(2**r**_5_ − 1)

26:   **if** **Y** < any of the best solutions **then**

27:    **Search** around current solution

28:   **else**

29:    **V**(*t* + 1) = **K**_3_*V*(*t*) + **K**_4_**r**_1_(**P**_*best*_(*t*) − **P**(*t*)) + **K**_5_**r**_2_(**P**_*Gbest*_ − **P**(*t*))

30:   **end if**

31:  **end for**

32:  **Get** solutions

33:  **Update** fitness

34: **end while**

35: **Return** best agent position **Y**

### 3.2 Exploitation group

This group is responsible for exploitation, which is the act of locating better spots near existing good solutions; to accomplish this, ADGWDTO employs two strategies.

#### 3.2.1 Moving towards the best solution

Using the following equation, the individual works toward the optimal solution:

#### 3.2.2 Search around the leader

The individuals search around the leader and that is because it increases the probability of obtaining a better solution ADGWDTO do that by using the following equations:

$$\begin{array}{c} D=L\left(t\right)*\left(K-r_{4}\right) \end{array}$$ (27)

$$\begin{array}{c} S\left(t+1\right)=S\left(t\right)+D.\left(2r_{5}-1\right) \end{array}$$ (28)

$$\begin{array}{c} K=1-\frac{2k*Xt^{2}}{Solutions-count^{2}} \end{array}$$ (29)

The the velocity of the agent, **V**(*t*+ 1), is calculated at iteration *i* + 1 as

$$\begin{array}{cc} V(t+1) & =K_{3}V\left(t\right)+K_{4}r_{1}(P_{best}\left(t\right)\\ & -P\left(t\right))+K_{5}r_{2}\left(P_{Gbest}-P\left(t\right)\right) \end{array}$$ (30)

where **P**_*best*_(*t*) is the best bird position. The **K**_1_, **K**_2_, and **K**_3_ are variable weights, while **K**_4_ and **K**_5_ are constants. **r**_1_ and **r**_2_ are randomly selected in [0, 1].

### 3.3 Adaptive dynamic approach

Fitness values are calculated for each solution in a population upon initialization of the optimization process. As a result of this, the best agent is selected by the optimization algorithm. The optimization algorithm begins the adaptive dynamic process by dividing the population of agents into the exploration group and exploitation group. The exploration group’s primary goal is to locate the leaders. The exploitation group’s primary goal is finding the best or most optimum solution. There is a constant exchange of information between the population groupings’ agents. The algorithm starts with a population with half its number in the exploration group and the other half in the exploitation group. The number of agents in each of the two groups should be balanced and dynamically changed throughout multiple iterations to acquire the best or most optimum solution.

### 3.4 Responsive exploration

The ADGWDTO starts populating using a variety of different solutions. The ADGWDTO calculates its best solution through the usage of the fitness function. And then, it divides the population into two separate groups, group A for exploration and group B for exploitation. In the beginning, the ADGWDTO divides the population by 70% for group A which is responsible for the exploration task. Group B takes 30% which is accountable for the exploitation task. As mentioned above, group A takes the most significant percentage at the beginning to accomplish the most incredible amount of search exploration. But what is to note is that this percentage changes dynamically during the iteration. As with each iteration, the ADGWDTO examines the convergence and best solution of the current iteration relative to the two preceding iterations. If the optimal solution has remained unchanged for three iterations in a row, the number of solutions in group A will be increased to facilitate exploration.

Moreover, this will help to avoid local optima. All this makes the ADGWDTO more responsive to the changes during the iteration to achieve the balance between exploring the search space and finding the good point around the best solution. This results in avoiding being caught in the local optimum and locating the most likely optimal solution.

### 3.5 Elitism

To guarantee the convergence quality throughout iterations, an elitism is added to the proposed ADGWDTO. Elitism allows the best agent from the current generation to carry over to the next, unaltered. This guarantees that the solution quality obtained by the ADGWDTO will not decrease from one generation to the next.

### 3.6 Exploration-exploitation balance

The ADGWDTO needs to strike a healthy balance between exploitation and exploration, and one way to do this is by regularly altering the population number. The algorithm starts by placing half of the population in the exploration group and the other half in the exploitation group. It then makes adjustments based on the results of these two groups’ activities. When doing the early rounds of the optimization process, it is helpful to have a significant proportion of individuals participating in the exploration group. This makes it easier to investigate the potentially fruitful areas of the search space. The number of people in the exploitation group continues to increase over time, while the number of people in the exploration group continues to decrease dynamically over time. This allows more people to improve their overall fitness by enabling more people in the exploitation group to improve their fitness. In addition to this, it uses elitism as a method to keep the process’s leader in consecutive populations, assuring convergence in the process in the event that a better solution cannot be found for those new populations. There is a possibility that the leader’s fitness will not improve significantly for the next three iterations in a row, which could lead to challenges with local optima and stagnation. As a direct result of this, ADGWDTO might increase the number of people in the exploratory group.

### 3.7 Binary optimizer

The output solution of the proposed ADGWDTO should be converted to binary [0, 1] for feature selection. The most common method to make this conversion is using the sigmoid function, which can convert an optimizer’s continuous solution to a binary solution.

$$\begin{array}{c} \begin{array}{cc} & S_{b}^{\left(t+1\right)}=\{\begin{array}{cc} 1 & \text{if}\;Sigmoid(S)\geq 0.5\\ 0 & otherwise \end{array},\\ & Sigmoid\left(S\right)=\frac{1}{1+e^{-10(S-0.5)}} \end{array} \end{array}$$ (31)

where *S* refers to the best position, and *t* is the iteration number. The phases of the proposed binary ADGWDTO algorithm are displayed in Algorithm 4.

**Algorithm 4**: The proposed binary bADGWDTO algorithm.

1: **Initialize** ADGWDTO parameters

2: **while** *t* ≤ *Max*_*iter*_ **do**

3:  **Apply** ADGWDTO algorithm

4:  **Find** best agent (*S*)

5:  **Get** the binary value of *S* using Eq (31)

6:  **Calculate** Fitness

7:  **Update** Positions and velocities of the best agents, *t* = *t* + 1

8: **end while**

9: **Return** best solution

### 3.8 Fitness function

The proposed optimization algorithm’s performance is evaluated using a fitness function. The fitness function is influenced by the selected features and the error rate of prediction. The selected features with reduced error rates and fewer features are a better example of a successful feature selection. In the suggested feature selection approach, the following fitness function is utilized.

$$\begin{array}{c} F_{n}=w_{1}Error\left(D\right)+w_{2}\frac{Num\;of\;selected\;features}{Total\;num\;of\;features} \end{array}$$ (32)

where *w*_1_ ∈ [0, 1] and *w*_2_ = 1 − *w*_1_ which are used to manage the significance of the number of selected features for a population of size *n* and the error rate of categorization.

If it is possible to give a subset of features that is capable of creating a low classification error rate, then the method can be considered adequate. The k-nearest neighbor technique, sometimes known as k-NN, is an easy classification method that is commonly used. The utilization of the k-nearest neighbors classifier in this method ensures that the characteristics that were selected are of high quality. The shortest distance between the query instance and the training examples is the only factor that is utilized in the process of determining classifiers. No model for the K-nearest neighbors is utilized in this experiment.

### 3.9 Complexity analysis

For population agents *n* and iterations *Max*_*iter*_, the complexity analysis of the ADGWDTO algorithm is expressed as follows.

- Initialization of population, **X**_*i*_(*i* = 1, 2, …, *n*), and various ADGWDTO algorithm parameters: *O*(1).
- Calculation of fitness function **F**_*n*_ for each agent **X**_*i*_: *O*(*n*).
- Find the best agent: *O*(*n*).
- Calculate **D** = **L**(*t*) * (**K** − **r**_4_): *Max*_*iter*_
- Calculate **S**(*t* + 1) = **S**(*t*) + **D**.(2**r**_5_ − 1): *Max*_*iter*_
- Update the number of agents in each group: *Max*_*iter*_
- Increase in the exploration group agents number: *Max*_*iter*_
- Mutate the solution: *Max*_*iter*_
- Update parameters: *Max*_*iter*_
- Decrease **k** exponentially from 1 to 0: *Max*_*iter*_
- Update positions of current agents in the exploration group: *O*(*t*_*max*_ × *n*).
- Get solutions: *Max*_*iter*_
- Update fitness: *Max*_*iter*_
- return the best agent position: *O*(1).

The complexity of computations from the above analysis is *O*(*Max*_*iter*_ × *n*) and *O*(*Max*_*iter*_ × *n* × *d*) with *d* dimension.

## 4 Experimental results

This section presents and discusses the experimental conditions and findings based on the proposed ADGWDTO algorithm for wind speed prediction. The section then covers the outcomes of three scenarios: feature selection, ensemble model, and comparison to rival methods. The proposed ADGWDTO algorithm is evaluated using benchmark functions F1 through F7 [32]. Appendix A displays the mean and standard deviation, convergence curves, ANOVA and T-test results for the benchmark functions.

### 4.1 Dataset

**Fig 2:**
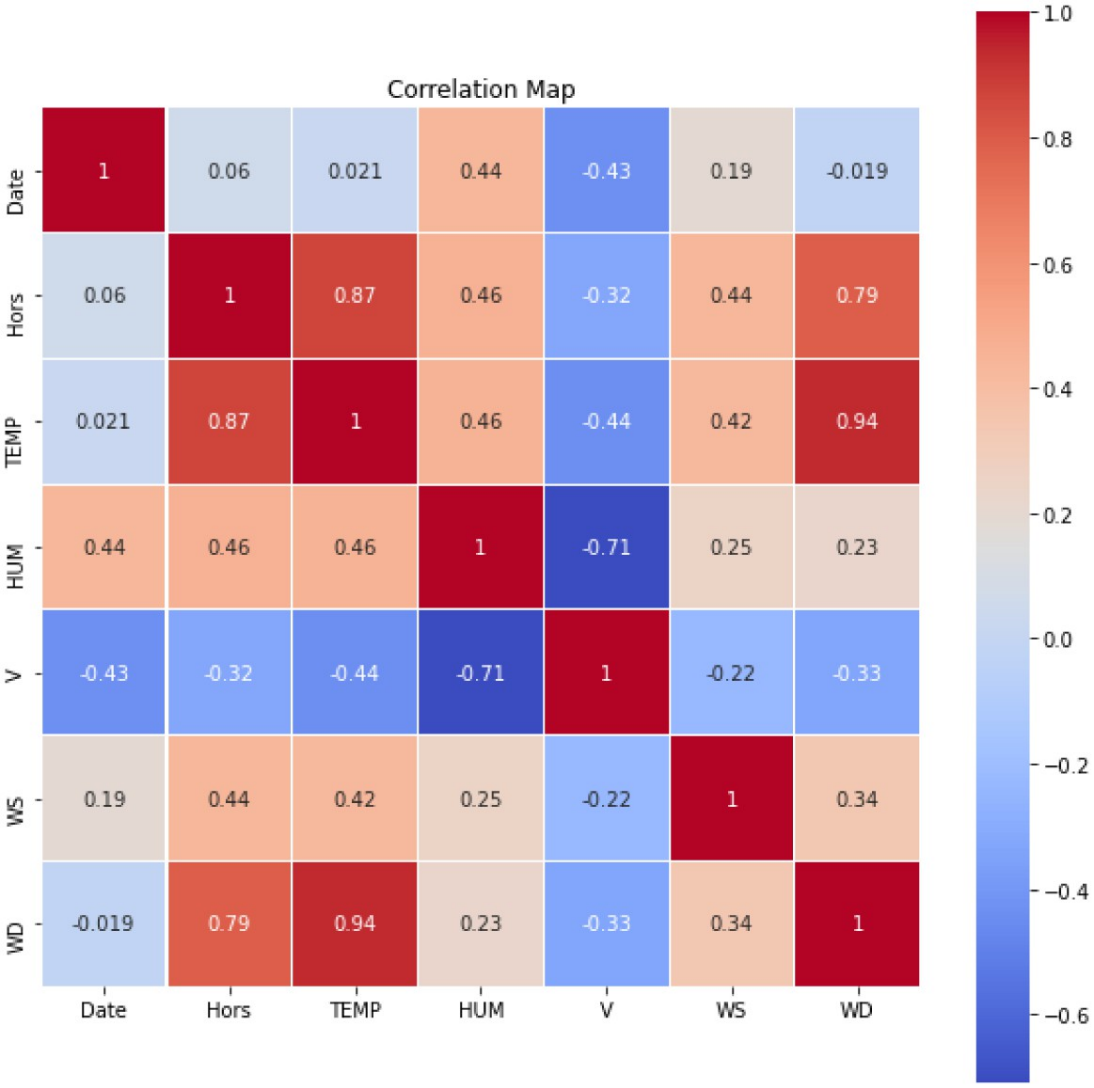
The correlation between the features of the dataset.

The tests are based on a dataset for wind power forecasting to anticipate the future hourly power generation at seven wind farms for up to 48 hours. The dataset used is titled “Global Energy Forecasting Competition 2012—Wind Forecasting” and is available on Kaggle [21]. This dataset contains seven features for seven wind farms, including wind speed and wind direction. The correlation between the features of the dataset is shown in Fig 2.

#### 4.1.1 Dataset preprocessing

As the recordings of the wind features might contain missing values of the wind features, it is crucial to preprocess the dataset before training the machine learning models. To deal with the missing values, the previous and next non-missing values are averaged and used to set the values of the lost recordings. On the other hand, scaling and normalizing dataset values are essential to guarantee that the machine learning model considers all features similarly. This article employs the min-max scaler as a fundamental data scaling approach, including scaling and bounding data features between 0 and 1. The following equation expresses the min-max scaler used in this article.

$$\begin{array}{c} X_{scaled}=\frac{X_{val}-X_{min}}{X_{max}-X_{min}} \end{array}$$ (33)

### 4.2 Evaluation criteria

The achieved results are assessed in terms of the criteria presented in Tables 2 and 3. The criteria listed in Table 2 are used to evaluate the performance of the proposed feature selection method, whereas the criteria listed in Table 3 are used to assess the prediction results achieved by the proposed algorithm. In addition, in these tables, the number of runs of the proposed and other competing optimizers is indicated as *M*, and $S_{j}^{*}$ represents the best agent at run number. $size(S_{j}^{*})$ indicates best solution vector size. *N* is the number of test set points. Predicted and actual values are represented by $\hat{V_{n}}$ and *V*_*n*_, respectively.

**Table 2:** Evaluation metrics used in assessing the proposed feature selection method.

Metric	Value
Mean	$\frac{1}{M}\sum_{i=1}^{M}S_{i}^{*}$
Best Fitness	$min_{i=1}^{M}S_{i}^{*}$
Worest Fitness	$max_{i=1}^{M}S_{i}^{*}$
Average fitness size	$\frac{1}{M}\sum_{i=1}^{M}size\left(S_{i}^{*}\right)$
Average Error	$\frac{1}{M}\sum_{j=1}^{M}\frac{1}{N}\sum_{i=1}^{N}mse\left(\hat{V_{i}}-V_{i}\right)$
Standard deviation	$\sqrt{\frac{1}{M-1}\sum_{i=1}^{M}{\left(S_{i}^{*}-Mean\right)}^{2}}$

**Table 3:** The evaluation metrics used in assessing the prediction results based on the proposed optimization algorithm.

Metric	Value
RMSE	$\sqrt{\frac{1}{N}\sum_{n=1}^{N}{\left(\hat{V_{n}}-V_{n}\right)}^{2}}$
RRMSE	$\frac{RMSE}{\sum_{n=1}^{N}\hat{V_{n}}}\times 100$
MAE	$\frac{1}{N}\sum_{n=1}^{N}\left|\hat{V_{n}}-V_{n}\right|$
MBE	$\frac{1}{N}\sum_{n=1}^{N}\left(\hat{V_{n}}-V_{n}\right)$
NSE	$1-\frac{\sum_{n=1}^{N}{\left(V_{n}-\hat{V_{n}}\right)}^{2}}{\sum_{n=1}^{N}{\left(V_{n}-\bar{\hat{V_{n}}}\right)}^{2}}$
WI	$1-\frac{\sum_{n=1}^{N}\left|\hat{V_{n}}-V_{n}\right|}{\sum_{n=1}^{N}|V_{n}-\bar{V_{n}}\left|+\right|\hat{V_{n}}-\bar{\hat{V_{n}}}|}$
*R* ^2^	$1-\frac{\sum_{n=1}^{N}{\left(V_{n}-\hat{V_{n}}\right)}^{2}}{\sum_{n=1}^{N}{\left(\sum_{n=1}^{N}V_{n})-V_{n}\right)}^{2}}$
r	$\frac{\sum_{n=1}^{N}\left(\hat{V_{n}}-\bar{\hat{V_{n}}}\right)\left(V_{n}-\bar{V_{n}}\right)}{\sqrt{\left(\sum_{n=1}^{N}{\left(\hat{V_{n}}-\bar{\hat{V_{n}}}\right)}^{2})(\sum_{n=1}^{N}{\left(V_{n}-\bar{V_{n}}\right)}^{2}\right)}}$

### 4.3 Feature selection results

This experiment aims to demonstrate the efficacy and efficiency of the proposed binary bADGWDTO algorithm for feature selection. The evaluation metrics offered in Table 2 are utilized to evaluate the outcomes attained by the proposed algorithm to those attained by competing methods, such as bGWO [19], bPSO [33], bGWOPSO [24], bGA [22], bGWOGA [34], binary bat algorithm (bBA) [35], bWOA [25], binary biogeography optimization (bBBO) [36], binary Multiverse Optimization (bMVO) [37], binary Satin Bowerbird Optimizer (bSBO) [38], and binary Firefly Algorithm (bFA) [39]. The configuration parameters of the proposed algorithm are listed in Table 4 and the configuration parameters of other algorithms are presented in Table 5. The evaluation of the results achieved by the suggested optimization approach and other competing methods is presented in Table 6. As shown in this table, the bADGWDTO algorithm offered achieves the best results compared to other approaches.

**Table 4:** Proposed algorithm configuration parameters.

Parameter	Value
Number of iterations	80
Number of search agents	20
Search domain	[0, 1]
Runs repetitions	20
Mutation rate	0.1
*h*_1_	0.99
*h*_2_	0.01

**Table 5:** Configuration parameters of the comparison algorithms.

Algorithm	Parameters	Values
GA	Selection method	Roulette wheel
	Crossover rate	0.9
	Mutation rate	0.1
FA	Fireflies count	10
PSO	*C*_1_, *C*_2_	[2, 2]
	*W*_*min*_, *W*_*max*_	[0.6, 0.9]
WOA	*a*	2 to 0
	*r*	[0, 1]
GWO	*a*	2 to 0

**Table 6:** Performance of proposed algorithm compared to state-of-the-art algorithms for feature selection.

	Avg. Error	Avg. Sel. Size	Avg. Fitness	Best Fitness	Worst Fitness	STD Fitness
bADGWDTO	0.8577	0.8105	0.9209	0.8227	0.9212	0.7432
bGWO	0.8749	1.0105	0.9371	0.8574	0.9243	0.7479
bGWO_PSO	0.9142	1.1438	0.9454	0.8989	1.0089	0.7661
bPSO	0.9087	1.0105	0.9355	0.9158	0.9835	0.7473
bBA	0.9183	1.1499	0.9584	0.8481	0.9497	0.7572
bWOA	0.9085	1.1739	0.9433	0.9074	0.9835	0.7495
bBBO	0.8769	1.1743	0.9412	0.9309	1.0174	0.7922
bSBO	0.917	1.1808	0.9752	0.9183	0.998	0.8082
bFA	0.9071	1.045	0.9874	0.9061	1.0037	0.7841
bGA	0.8885	0.9529	0.9485	0.8518	0.9669	0.7495

### 4.4 Ensemble prediction results

The features selected by the proposed bADGWDTO are used to train a new ensemble model composed of three regression models: MLP, KNR, and LSTM. The participation of the prediction results generated by these regression models in predicting the final value of the wind speed is weighted and optimized using the proposed ADGWDTO. The weights of the three regression models are optimized using the proposed optimizer and then averaged to generate the final results. Table 7 presents the evaluation results of the proposed ensemble using ADGWDTO with comparison to the base regression models and two other ensemble models, namely, average ensemble and ensemble using support vector regression (SVR). The table shows that the proposed optimized ensemble achieves the best results when measured based on all the evaluation criteria presented earlier. These results confirm the superiority of the proposed approach in predicting the wind speed more robustly.

**Table 7:** Performance of the proposed weighted optimized ensemble model compared to state-of-the-art algorithms.

	MLP Regressor	LSTM	K-Neighbors Regressor	Average Ensemble	Ensemble using SVR	Ensemble using ADGWDTO
RMSE	0.150217182	0.017911248	0.157801477	0.100417713	0.010771429	0.003478279
MAE	0.123015941	0.003278629	0.12705746	0.081873329	0.002029839	0.002098412
MBE	0.002593856	-9.96E-05	0.017277218	0.0065905	-0.00051129	-5.80E-05
r	0.611041524	0.995530462	0.561788402	0.889059564	0.998391236	0.9998598
*R* ^2^	0.373371745	0.991080901	0.315606209	0.790426908	0.99678506	0.999719619
RRMSE	35.36854105	4.217192	37.15425861	37.15425861	2.536126082	0.641014509
NSE	0.371953399	0.991070969	0.306933707	0.719344664	0.996770768	0.999719067
WI	0.602783729	0.989413366	0.589733738	0.735632487	0.993445687	0.994214272

In order to demonstrate the efficacy of the suggested optimization algorithm, the proposed ensemble model is optimized using GA, FA, PSO, WOA, GWO, and DTO in addition to the proposed optimization technique. The optimized ensemble model results are shown in Table 8. This table displays an analysis of the outcomes obtained by the optimizers-based optimized ensemble. In the first column of the table are the outcomes of the proposed method. These results demonstrate robust and superior performance in comparison to the optimization ensembles of other optimizers. These results demonstrate that the suggested optimization procedure is superior to previous methods for determining the optimal ensemble model parameters.

**Table 8:** Analysis of the results achieved by the proposed ensemble model compared to state-of-the-art algorithms.

	ADGWDTO	DTO	GWO	WOA	PSO	FA	GA
Number of values	20	20	20	20	20	20	20
Range	0.0003	0.002	0.00209	0.003	0.002	0.0029	0.003102
Coefficient of variation	1.470%	5.983%	5.078%	8.272%	4.579%	6.456%	7.187%
25% Percentile	0.003478	0.005423	0.006678	0.009987	0.007745	0.008569	0.009998
75% Percentile	0.003478	0.005423	0.006678	0.009987	0.007745	0.008569	0.009998
Minimum	0.003278	0.004423	0.005678	0.006987	0.006745	0.007057	0.006998
Mean	0.003473	0.005423	0.006683	0.009687	0.007793	0.008472	0.009798
Median	0.003478	0.005423	0.006678	0.009987	0.007745	0.008569	0.009998
Maximum	0.003578	0.006423	0.007768	0.009987	0.008745	0.009957	0.0101
STD Error of Mean	0.00001141	0.00007255	0.00007588	0.0001792	0.0000798	0.0001223	0.0001575
STD Deviation	0.00005104	0.0003244	0.0003393	0.0008013	0.0003569	0.000547	0.0007042
Geometric SD factor	1.015	1.063	1.052	1.098	1.047	1.068	1.086
Geometric mean	0.003473	0.005414	0.006674	0.00965	0.007785	0.008455	0.009769
Sum	0.06947	0.1085	0.1337	0.1937	0.1559	0.1694	0.196

### 4.5 Statistical analysis

To prove the stability and significance of the proposed algorithm, two types of statistical tests were performed, namely the one-way analysis of variance (ANOVA) test and the Wilcoxon rank-sum test. In the ANOVA test, the mean, *μ*, values of null hypothesis represented by *H*0 includes *μ*ADGWDTO = *μ*GA = *μ*FA = *μ*PSO = *μ*WOA = *μ*GWO = *μ*DTO. Table 9 displays the ANOVA test’s measured values. Using the Wilcoxon rank-sum test, the p-values of the proposed ADGWDTO algorithm are compared to those of alternative optimization techniques. Using the statistical difference between each pair of competing algorithms, the p-values between the proposed ADGWDTO and the other competing algorithms are computed to demonstrate that the proposed technique is significantly distinct. This analysis is carried out using Wilcoxon’s rank-sum test in which the mean, *μ*, values of null hypothesis is represented by *H*0 includes *μ*ADGWDTO = *μ*GA, *μ*ADGWDTO = *μ*FA, *μ*ADGWDTO = *μ*PSO, *μ*ADGWDTO = *μ*WOA, *μ*GWO = *μ*ADGWDTO, and *μ*ADGWDTO = *μ*DTO. The *H*1 hypothesis, on the other hand, does not compare the means of the algorithms. The results of the Wilcoxon rank-sum test are presented in the Wilcoxon Table 10. Between the proposed approach and other algorithms, the p-values are less than 0.05. These results demonstrate the statistical relevance of the suggested optimization procedure.

**Table 9:** ANOVA test results for the proposed algorithm versus state-of-the-art algorithms.

	SS	DF	MS	F (DFn, DFd)	P value
Residual (within columns)	0.00003396	133	2.554E-07		
Treatment (between columns)	0.0006419	6	0.000107	F (6, 133) = 419.0	P<0.0001
Total	0.0006759	139			

**Table 10:** Wilcoxon signed rank test results of the proposed algorithm compared to state-of-the-art algorithms.

	ADGWDTO	DTO	GWO	WOA	PSO	FA	GA
Number of values	20	20	20	20	20	20	20
Sum of positive ranks	210	210	210	210	210	210	210
Sum of negative ranks	0	0	0	0	0	0	0
Sum of signed ranks (W)	210	210	210	210	210	210	210
Actual median	0.003478	0.005423	0.006678	0.009987	0.007745	0.008569	0.009998
P value (two tailed)	<0.0001	<0.0001	<0.0001	<0.0001	<0.0001	<0.0001	<0.0001
Theoretical median	0	0	0	0	0	0	0
Discrepancy	0.003478	0.005423	0.006678	0.009987	0.007745	0.008569	0.009998
Significant (alpha = 0.05)?	Yes	Yes	Yes	Yes	Yes	Yes	Yes
Exact or estimate?	Exact	Exact	Exact	Exact	Exact	Exact	Exact

### 4.6 Visual results

**Fig 3:**
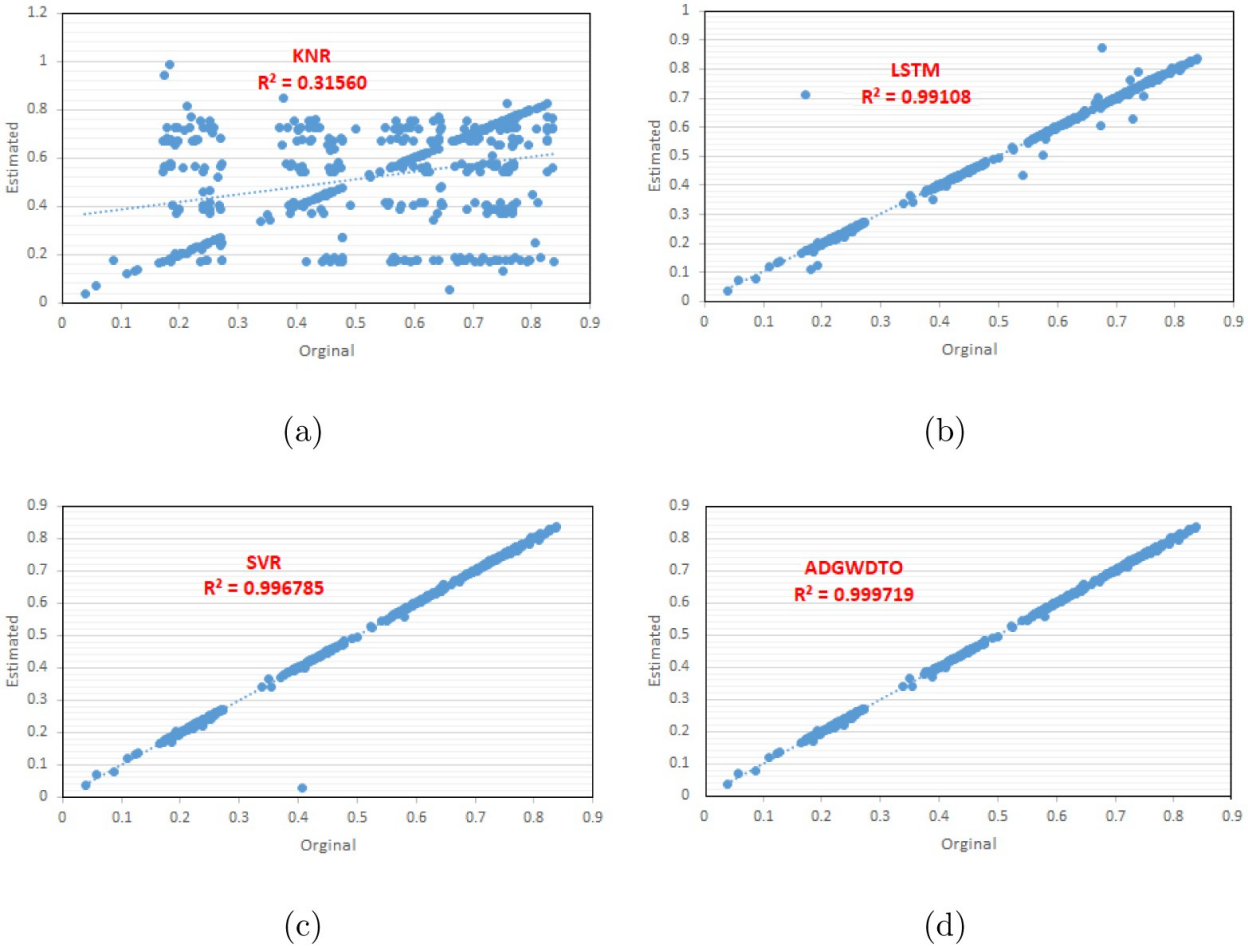
Fitting the prediction values resulting by KNR, LSTM, SVR, and the proposed ADGWDTO.

Fig 3 shows the predicted and actual wind speed values mapping by using the proposed weighted optimized ensemble models and the three base regression models. The figure shows that the proposed approach’s results fit a high accuracy line. However, fitting the other mapping fit line with distracted points affects the accuracy of the regression model. Therefore, the proposed approach results can be considered more accurate than the other methods.

**Fig 4:**
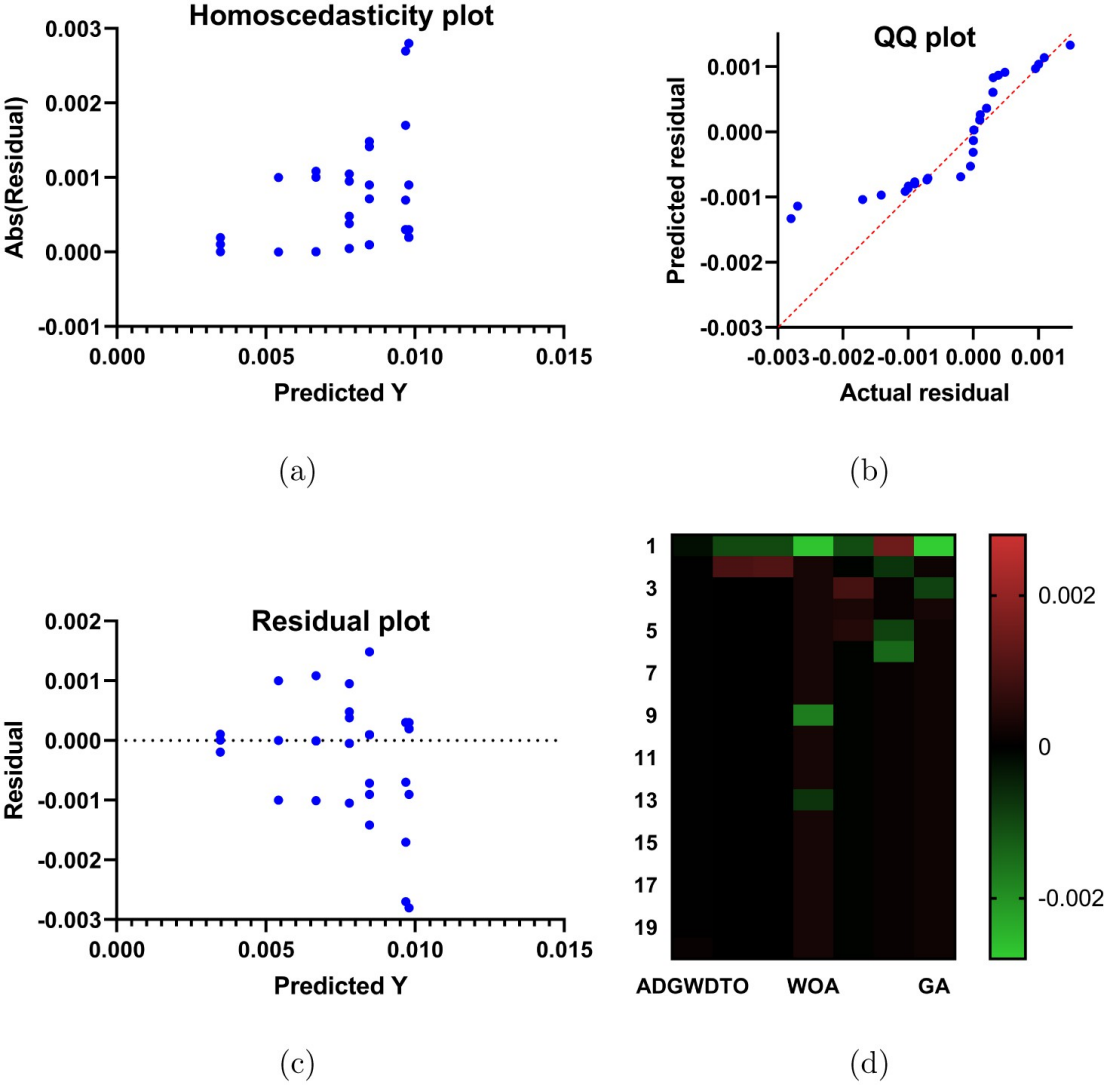
Plots and heat map of the ADGWDTO compared to state-of-the-art algorithms, (a) Homoscedasticity plot, (b) QQ plot, (c) Residual plot, and (d) Heat map.

**Fig 5:**
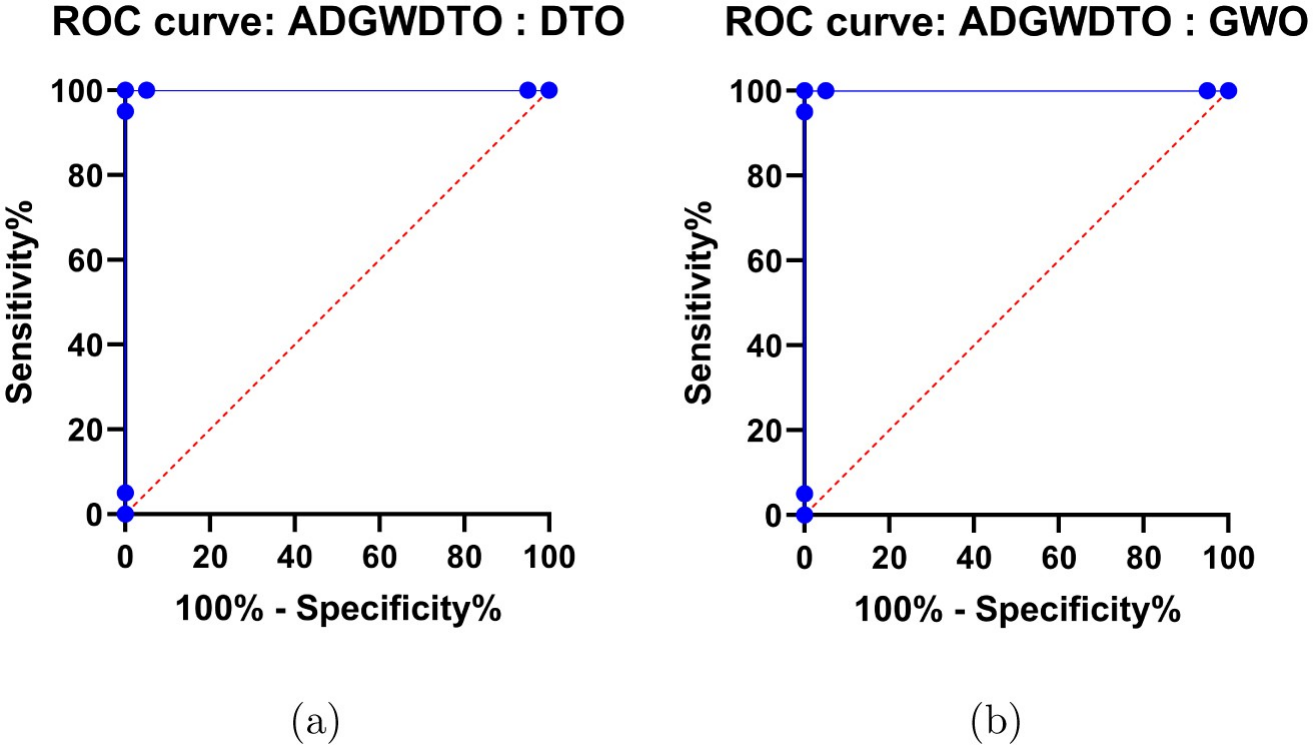
The ADGWDTO algorithm versus DTO and GWO algorithms ROC curves, (a) ADGWDTO versus DTO and (b) ADGWDTO versus GWO.

**Fig 6:**
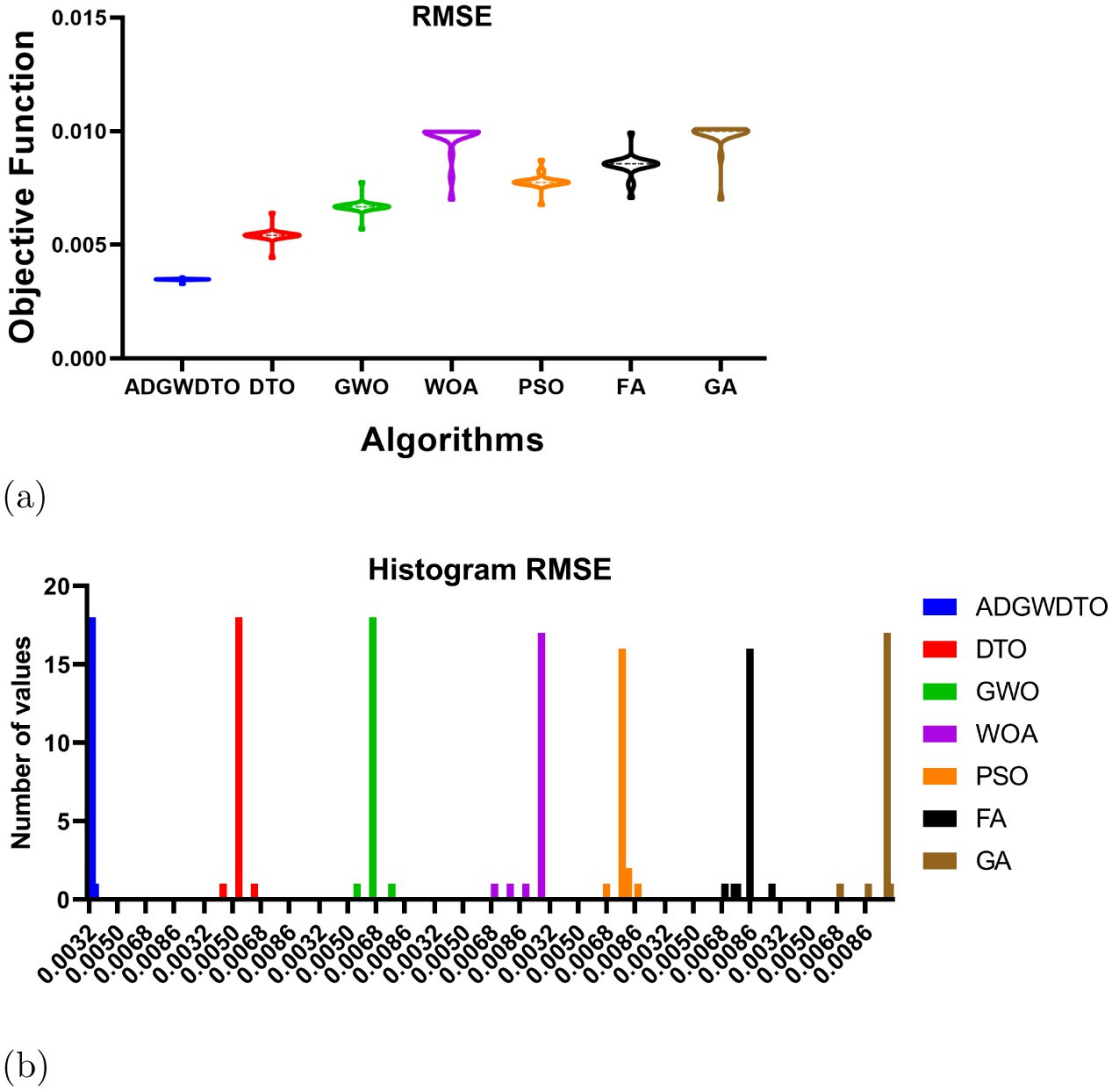
RMSE and Histogram RMSE of ADGWDTO algorithm compared to DTO, GWO, WOA, PSO, FA, and GA algorithms.

Figs 4–6 depict a series of visual plots representing the residual, homoscedasticity, and QQ, ROC, Heatmap, RMSE, and histogram of RMSE, respectively. The residual error lies within the range of -0.02 to +0.02 and the homoscedasticity values lie within the range of -0.001 to +0.003, demonstrating the robustness of the suggested method. In addition, the QQ plot demonstrates that the projected results match the actual values, validating the robustness of the suggested method. The ROC curves illustrate the maximum area under the curve attained by the suggested approach versus DTO and GWO. In addition, the heatmap and RMSE graphs demonstrate that the proposed optimization approach is superior.

Moreover, the histogram RMSE plot shows the number of RMSE values achieved by the proposed optimization algorithm and other optimization methods. It can be noted from this figure that the smallest RMSE values are performed by the proposed approach with the highest number of occurrences. These plots emphasized the findings previously discussed and clearly show the effectiveness and superiority of the proposed method.

## 5 Conclusion

A new meta-heuristic optimization-based method for improving the parameters of a weighted average ensemble model for forecasting wind speed in wind farms is presented in this paper. Through a mixture of the grey wolf optimizer and dipper throated optimization algorithms, the suggested algorithm achieves a better balance between exploitation and exploration groups of the optimization process. As a case study to demonstrate the efficacy of the proposed algorithm, the Kaggle dataset for wind power forecasting is used to estimate the hourly wind speed for the following 48 hours. Alternatively, a novel binary ADGWDTO algorithm is proposed to choose the significant features for improving the accuracy of prediction. Comparisons are made between the performance of the suggested algorithms and that of other feature selection techniques. The second series of experiments are done to compare the performance of the optimization algorithm against that of various regression and ensemble models. The comparison experiments contain two more ensembles, the average and support vector regression-based ensemble models. In addition, statistical analysis employing ANOVA and Wilcoxon’s rank-sum tests is conducted to confirm the importance of the proposed method. The experimental results based on several evaluation criteria proved the proposed method’s effectiveness, superiority, and robustness compared to state-of-art optimization approaches. The potential future work can be including various datasets to emphasize the generalization of the proposed algorithms for other fields such as Constrained engineering, classification, and feature selection challenges. Multiple approaches, such as sparse auto-encoding, can be compared with the proposed model in future work.

## 6 Appendix

**Fig 7:**
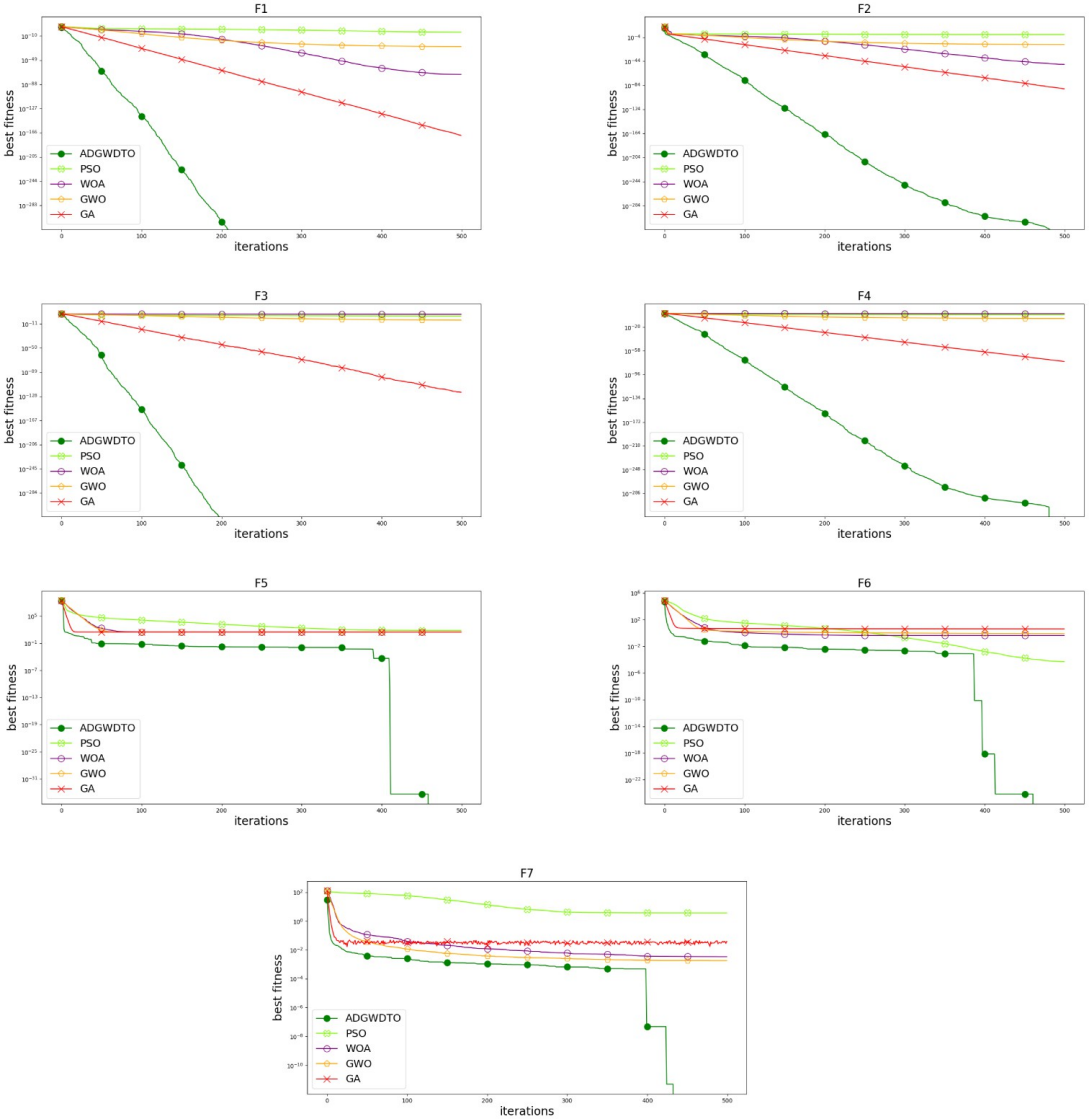
Proposed algorithm convergence curves versus other competing algorithms for the seven benchmark functions.

**Fig 8:**
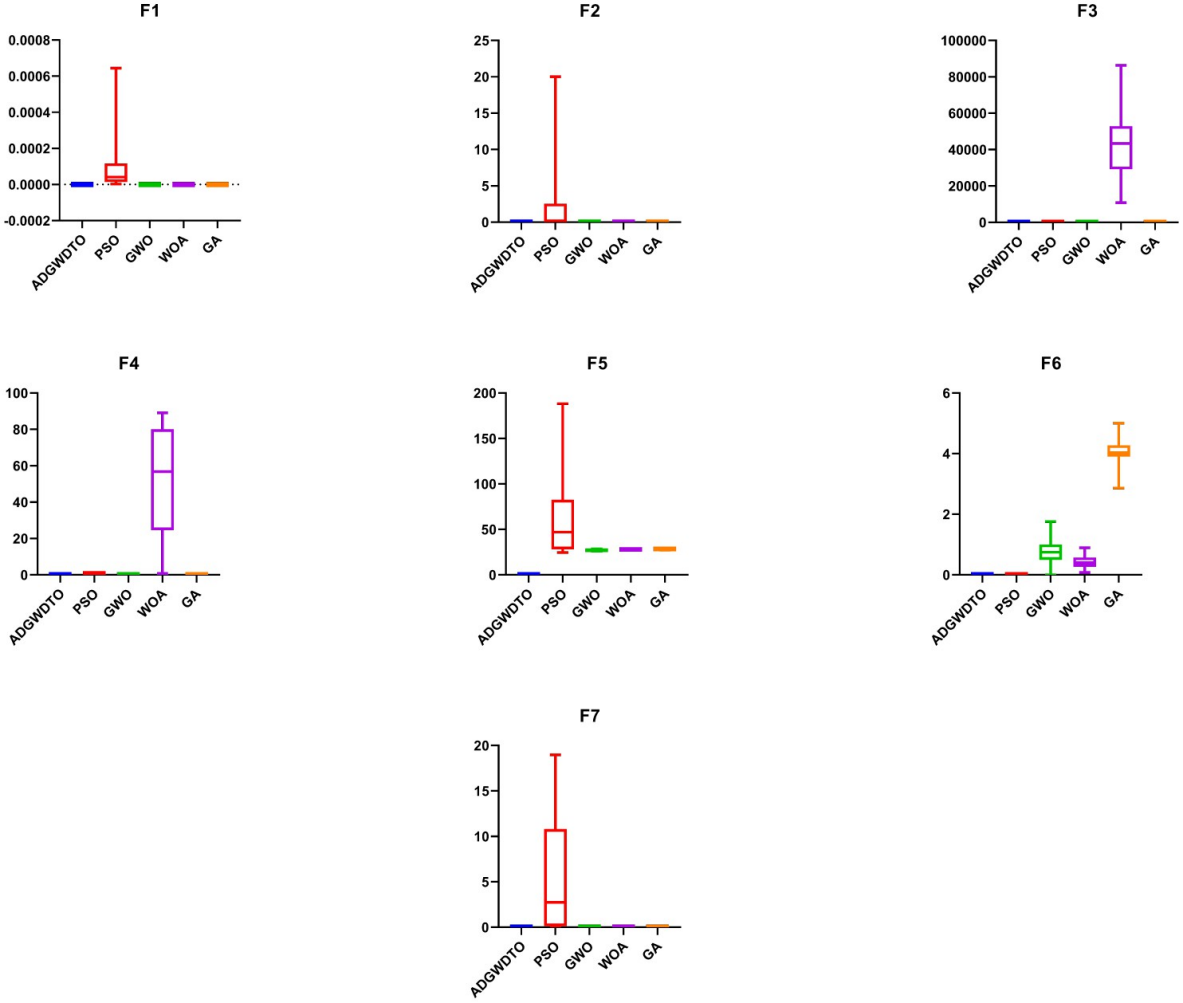
Box plot of the ADGWDTO and competing algorithms for the seven benchmark functions.

ADGWDTO is assessed for benchmark functions (F1 through F7) [32] in this appendix, as indicated in Table 11. Fig 7 compares the algorithm’s convergence curves to those of competing algorithms for benchmark functions. The rapid convergence of the suggested algorithm, as seen in this figure, demonstrates how the suggested approach improves the capability of exploration. Fig 8 is a box plot comparing the proposed method to competing algorithms for the seven benchmark functions. Table 12 presents the mean and standard deviation of the recommended and compared benchmark function algorithms (F1 to F7). The outcomes of the ANOVA test for the reference functions are shown in Table 13. The T-test for the benchmark functions (F1 through F7) using the recommended algorithm versus the compared techniques is presented in Table 14. The results illustrate the efficacy of the proposed methodology.

**Table 11:** Unimodal benchmark functions (F1 to F7).

Function	D	Range
$\text{F}1\left(w\right)=\sum_{i=1}^{n}w^{2}\;$	30	[-100, 100]
$\text{F}2\left(w\right)=\sum_{i=1}^{n}|w_{i}|+\prod_{i=1}^{n}\left|w_{i}\right|$	30	[-10, 10]
$\text{F}3\left(w\right)=\sum_{i=1}^{n}{\left(\sum_{j=1}^{i}w_{i}\right)}^{2}$	30	[-100, 100]
F4(*w*) = *max*_*i*_{|*w*_*i*_|, 1 ≤ *i* ≤ *D*}	30	[-100, 100]
$\text{F}5\left(w\right)=\sum_{i=1}^{D-1}\left[100{\left(w_{i+1}-w_{i}^{2}\right)}^{2}-{\left(w_{i}-1\right)}^{2}\right]$	30	[-30, 30]
$\text{F}6\left(w\right)=\sum_{i=1}^{D}{\left(w_{i}+0.5\right)}^{2}$	30	[-100, 100]
$\text{F}7\left(w\right)=\sum_{i=1}^{D}iw_{i}^{4}+rand\left[0,1\right]$	30	[-1.28, 1.28]

**Table 12:** Proposed algorithm mean and standard deviation (StDev) versus compared algorithms for the benchmark functions.

Func	Algorithm	ADGWDTO	PSO	WOA	GWO	GA
F1	Mean	0	0.000136	1.41 ×10^30^	6.59 ×10^−28^	4.6 ×10^−172^
	StDev	0	0.000202	4.91 ×10^−30^	6.34 ×10^−05^	0
F2	Mean	0	0.042144	1.06 ×10^−21^	7.18 ×10^−17^	3.44 ×10^−90^
	StDev	0	0.045421	2.39 ×10^−21^	0.029014	6.13 ×10^−90^
F3	Mean	0	70.12562	5.39 ×10^−07^	3.29 ×10^−06^	1.7 ×10^−127^
	StDev	0	22.11924	2.93 ×10^−06^	79.14958	8.6 ×10^−127^
F4	Mean	0	1.086481	0.072581	5.61 ×10^−07^	1.15 ×10^−75^
	StDev	0	0.317039	0.39747	1.315088	2.45 ×10^−75^
F5	Mean	0	96.71832	27.86558	26.81258	28.37287
	StDev	0	60.11559	0.763626	69.90499	0.582802
F6	Mean	0	0.000102	3.116266	0.816579	3.932626
	StDev	0	8.28 ×10^−05^	0.532429	0.000126	0.431755
F7	Mean	0	0.122854	0.001425	0.002213	0.022992
	StDev	0	0.044957	0.001149	0.100286	0.021966

**Table 13:** ANOVA test over the seven benchmark functions, treatment is between columns and residual is within columns.

F1					
	SS	DF	MS	F (DFn, DFd)	P value
Treatment	2.42E-07	4	6.05E-08	F (4, 145) = 12.04	P<0.0001
Residual	7.29E-07	145	5.03E-09		
Total	9.71E-07	149			
F2					
	SS	DF	MS	F (DFn, DFd)	P value
Treatment	269.7	4	67.43	F (4, 145) = 7.730	P<0.0001
Residual	1265	145	8.723		
Total	1535	149			
F3					
	SS	DF	MS	F (DFn, DFd)	P value
Treatment	4.25E+10	4	1.06E+10	F (4, 145) = 186.8	P<0.0001
Residual	8.24E+09	145	56830905		
Total	5.07E+10	149			
F4					
	SS	DF	MS	F (DFn, DFd)	P value
Treatment	61550	4	15387	F (4, 145) = 92.40	P<0.0001
Residual	24147	145	166.5		
Total	85697	149			
F5					
	SS	DF	MS	F (DFn, DFd)	P value
Treatment	59376	4	14844	F (4, 145) = 42.46	P<0.0001
Residual	50691	145	349.6		
Total	110067	149			
F6					
	SS	DF	MS	F (DFn, DFd)	P value
Treatment	355.2	4	88.79	F (4, 145) = 1261	P<0.0001
Residual	10.21	145	0.07039		
Total	365.4	149			
F7					
	SS	DF	MS	F (DFn, DFd)	P value
Treatment	566.5	4	141.6	F (4, 145) = 16.82	P<0.0001
Residual	1221	145	8.422		
Total	1788	149			

**Table 14:** Benchmark functions T-test for the proposed and compared algorithms.

	ADGWDTO vs PSO	ADGWDTO vs GWO	ADGWDTO vs WOA	ADGWDTO vs GA
F1	2.22 ×10^−05^	2.22 ×10^−05^	2.22 ×10^−05^	2.22 ×10^−05^
F2	2.22 ×10^−05^	2.22 ×10^−05^	2.22 ×10^−05^	2.22 ×10^−05^
F3	2.22 ×10^−05^	2.22 ×10^−05^	2.22 ×10^−05^	2.22 ×10^−05^
F4	2.22 ×10^−05^	2.22 ×10^−05^	2.22 ×10^−05^	2.22 ×10^−05^
F5	2.22 ×10^−05^	2.22 ×10^−05^	2.22 ×10^−05^	2.22 ×10^−05^
F6	2.22 ×10^−05^	2.22 ×10^−05^	2.22 ×10^−05^	2.22 ×10^−05^
F7	2.22 ×10^−05^	2.22 ×10^−05^	2.22 ×10^−05^	2.22 ×10^−05^

## References

[1] Santhosh M, Venkaiah C, Vinod Kumar DM. Current advances and approaches in wind speed and wind power forecasting for improved renewable energy integration: A review. Engineering Reports. 2020;2(6):e12178. doi: 10.1002/eng2.12178

[2] Kosovic B, Haupt SE, Adriaansen D, Alessandrini S, Wiener G, Delle Monache L, et al. A Comprehensive Wind Power Forecasting System Integrating Artificial Intelligence and Numerical Weather Prediction. Energies. 2020;13(6). doi: 10.3390/en13061372

[3] Lin Z, Liu X, Collu M. Wind power prediction based on high-frequency SCADA data along with isolation forest and deep learning neural networks. International Journal of Electrical Power & Energy Systems. 2020;118:105835. doi: 10.1016/j.ijepes.2020.105835

[4] Ibrahim M, Alsheikh A, Al-Hindawi Q, Al-Dahidi S, ElMoaqet H. Short-Time Wind Speed Forecast Using Artificial Learning-Based Algorithms. Computational intelligence and neuroscience. 2020;2020:8439719. doi: 10.1155/2020/8439719 32377179 PMC7197004

[5] Lima J, Guetter A, Freitas S, Panetta J, de Mattos JG. A Meteorological–Statistic Model for Short-Term Wind Power Forecasting. Journal of Control, Automation and Electrical Systems. 2017;28(5):679–691. doi: 10.1007/s40313-017-0329-8

[6] Osorio GJ, Matias JCO, Catalão JPS. Short-term wind power forecasting using adaptive neuro-fuzzy inference system combined with evolutionary particle swarm optimization, wavelet transform and mutual information. Renewable Energy. 2015;75:301–307. doi: 10.1016/j.renene.2014.09.058

[7] Khodayar M, Wang J, Manthouri M. Interval Deep Generative Neural Network for Wind Speed Forecasting. IEEE Transactions on Smart Grid. 2019;10(4):3974–3989. doi: 10.1109/TSG.2018.2847223

[8] Khodayar M, Kaynak O, Khodayar ME. Rough Deep Neural Architecture for Short-Term Wind Speed Forecasting. IEEE Transactions on Industrial Informatics. 2017;13(6):2770–2779. doi: 10.1109/TII.2017.2730846

[9] Khodayar M, Wang J. Spatio-Temporal Graph Deep Neural Network for Short-Term Wind Speed Forecasting. IEEE Transactions on Sustainable Energy. 2019;10(2):670–681. doi: 10.1109/TSTE.2018.2844102

[10] Jalali SM, Ahmadian S, Khodayar M, Khosravi A, Ghasemi V, Shafie-khah M, et al. Towards novel deep neuroevolution models: chaotic levy grasshopper optimization for short-term wind speed forecasting. Engineering with Computers. 2021;2021:1–25.

[11] Dhiman HS, Deb D, Guerrero JM. Hybrid machine intelligent SVR variants for wind forecasting and ramp events. Renewable and Sustainable Energy Reviews. 2019;108:369–379. doi: 10.1016/j.rser.2019.04.002

[12] Dhiman HS, Deb D. Machine intelligent and deep learning techniques for large training data in short-term wind speed and ramp event forecasting. International Transactions on Electrical Energy Systems. 2021;31(9):e12818. doi: 10.1002/2050-7038.12818

[13] Dhiman HS, Deb D, Muyeen SM, Kamwa I. Wind Turbine Gearbox Anomaly Detection Based on Adaptive Threshold and Twin Support Vector Machines. IEEE Transactions on Energy Conversion. 2021;36(4):3462–3469. doi: 10.1109/TEC.2021.3075897

[14] Wang J, Yang W, Du P, Li Y. Research and application of a hybrid forecasting framework based on multi-objective optimization for electrical power system. Energy. 2018;148:59–78. doi: 10.1016/j.energy.2018.01.112

[15] Bilal B, Ndongo M, Adjallah KH, Sava A, Kebe CMF, Ndiaye PA, et al. Wind turbine power output prediction model design based on artificial neural networks and climatic spatiotemporal data. In: 2018 IEEE International Conference on Industrial Technology (ICIT); 2018. p. 1085–1092.

[16] Zhang J, Yan J, Infield D, Liu Y, sang Lien F. Short-term forecasting and uncertainty analysis of wind turbine power based on long short-term memory network and Gaussian mixture model. Applied Energy. 2019;241:229–244. doi: 10.1016/j.apenergy.2019.03.044

[17] Hong YY, Rioflorido CLPP. A hybrid deep learning-based neural network for 24-h ahead wind power forecasting. Applied Energy. 2019;250:530–539. doi: 10.1016/j.apenergy.2019.05.044

[18] Ibrahim A, Mirjalili S, El-Said M, Ghoneim SSM, Al-Harthi MM, Ibrahim TF, et al. Wind Speed Ensemble Forecasting Based on Deep Learning Using Adaptive Dynamic Optimization Algorithm. IEEE Access. 2021;9:125787–125804. doi: 10.1109/ACCESS.2021.3111408

[19] El-kenawy ES, Eid M. HYBRID GRAY WOLF AND PARTICLE SWARM OPTIMIZATION FOR FEATURE SELECTION. International journal of innovative computing, information & control: IJICIC. 2020;16(3):831–844.

[20] Takieldeen AE, El-kenawy ESM, Hadwan M, Zaki RM. Dipper Throated Optimization Algorithm for Unconstrained Function and Feature Selection. Computers, Materials & Continua. 2022;72(1):1465–1481. doi: 10.32604/cmc.2022.026026

[21] Global Energy Forecasting Competition 2012—Wind Forecasting. https://www.kaggle.com/c/GEF2012-wind-forecasting Accessed: 2022-08-01

[22] Kabir MM, Shahjahan M, Murase K. A new local search based hybrid genetic algorithm for feature selection. Neurocomputing. 2011;74(17):2914–2928. doi: 10.1016/j.neucom.2011.03.034

[23] Fister I, Yang XS, Brest J, Fister IJ. 4—Memetic Self-Adaptive Firefly Algorithm. In: Yang XS, Cui Z, Xiao R, Gandomi AH, Karamanoglu M, editors. Swarm Intelligence and Bio-Inspired Computation. Oxford: Elsevier; 2013. p. 73–102.

[24] Bello R, Gomez Y, Nowe A, Garcia MM. Two-Step Particle Swarm Optimization to Solve the Feature Selection Problem. In: Seventh International Conference on Intelligent Systems Design and Applications (ISDA 2007); 2007. p. 691–696.

[25] Mirjalili S, Lewis A. The Whale Optimization Algorithm. Advances in Engineering Software. 2016;95:51–67. doi: 10.1016/j.advengsoft.2016.01.008

[26] Hassib EM, El-Desouky AI, Labib LM, El-Kenawy ESMT. WOA+BRNN: An imbalanced big data classification framework using Whale optimization and deep neural network. Soft Computing. 2020;24(8):5573–5592. doi: 10.1007/s00500-019-03901-y

[27] El-Kenawy ESM, Ibrahim A, Mirjalili S, Eid MM, Hussein SE. Novel Feature Selection and Voting Classifier Algorithms for COVID-19 Classification in CT Images. IEEE Access. 2020;8:179317–179335. doi: 10.1109/ACCESS.2020.3028012 34976558 PMC8545288

[28] Nazir MS, Alturise F, Alshmrany S, Nazir HMJ, Bilal M, Abdalla AN, et al. Wind Generation Forecasting Methods and Proliferation of Artificial Neural Network: A Review of Five Years Research Trend. Sustainability. 2020;12(9). doi: 10.3390/su12093778

[29] El-Kenawy ESM, Mirjalili S, Ibrahim A, Alrahmawy M, El-Said M, Zaki RM, et al. Advanced Meta-Heuristics, Convolutional Neural Networks, and Feature Selectors for Efficient COVID-19 X-Ray Chest Image Classification. IEEE Access. 2021;9:36019–36037. doi: 10.1109/ACCESS.2021.3061058 34812381 PMC8545230

[30] Nasser AA, Rashad MZ, Hussein SE. A Two-Layer Water Demand Prediction System in Urban Areas Based on Micro-Services and LSTM Neural Networks. IEEE Access. 2020;8:147647–147661. doi: 10.1109/ACCESS.2020.3015655

[31] Al-Hajj R, Assi A, Fouad MM. Stacking-Based Ensemble of Support Vector Regressors for One-Day Ahead Solar Irradiance Prediction. In: 2019 8th International Conference on Renewable Energy Research and Applications (ICRERA); 2019. p. 428–433.

[32] Ibrahim A, Ali HA, Eid MM, El-kenawy ESM. Chaotic Harris Hawks Optimization for Unconstrained Function Optimization. In: 2020 16th International Computer Engineering Conference (ICENCO). IEEE; 2020. Available from: 10.1109/icenco49778.2020.9357403.

[33] Şenel FA, Gökçe F, Yüksel AS, Yigit T. A novel hybrid PSO–GWO algorithm for optimization problems. Engineering with Computers. 2019;35(4):1359–1373. doi: 10.1007/s00366-018-0668-5

[34] El-Kenawy ESM, Eid MM, Saber M, Ibrahim A. MbGWO-SFS: Modified Binary Grey Wolf Optimizer Based on Stochastic Fractal Search for Feature Selection. IEEE Access. 2020;8:107635–107649. doi: 10.1109/ACCESS.2020.3001151

[35] Mugemanyi S, Qu Z, Rugema FX, Dong Y, Bananeza C, Wang L. Optimal Reactive Power Dispatch Using Chaotic Bat Algorithm. IEEE Access. 2020;8:65830–65867. doi: 10.1109/ACCESS.2020.2982988

[36] Zhang X, Wang D, Chen H. Improved Biogeography-Based Optimization Algorithm and Its Application to Clustering Optimization and Medical Image Segmentation. IEEE Access. 2019;7:28810–28825. doi: 10.1109/ACCESS.2019.2901849

[37] Mirjalili S, Mirjalili SM, Hatamlou A. Multi-Verse Optimizer: A Nature-Inspired Algorithm for Global Optimization. Neural Comput Appl. 2016;27(2):495–513. doi: 10.1007/s00521-015-1870-7

[38] Samareh Moosavi SH, Khatibi Bardsiri V. Satin bowerbird optimizer: A new optimization algorithm to optimize ANFIS for software development effort estimation. Engineering Applications of Artificial Intelligence. 2017;60:1–15. doi: 10.1016/j.engappai.2017.01.006

[39] Fister I, Yang XS, Fister I, Brest J. Memetic firefly algorithm for combinatorial optimization. Bioinspired Optimization Methods and Their Applications-BIOMA. 2012; p. 75–86.

